# Silicon (Si) foliar treatment modulates *Capsicum annuum* L. (green chilli) growth and stress responses under cadmium and lead stress

**DOI:** 10.3389/fpls.2025.1590148

**Published:** 2025-06-13

**Authors:** Waquar Akhtar Ansari, Mohammad Shahid, Mohammad Danish, Sajad Ali, Mohammed A. Almalki, Mohammad Alfredan

**Affiliations:** ^1^ Marwadi University Research Center, Department of Agriculture, Faculty of Science, Marwadi University, Rajkot, Gujarat, India; ^2^ Department of Agricultural Microbiology, Faculty of Agriculture Science, Aligarh Muslim University (A.M.U.), Aligarh, Uttar Pradesh, India; ^3^ Botany Section, School of Sciences, Maulana Azad National Urdu University, Hyderabad, India; ^4^ Department of Biological Sciences, College of Science, King Faisal University, Al-Ahsa, Saudi Arabia

**Keywords:** heavy metals, silicon (Si), resilience mechanism, metal uptake, stress responsive gene

## Abstract

Silicon (Si) plays a crucial role in improving plant resilience against abiotic stresses including heavy metals (HMs). However, little is known about its role in chilli plants during HM stresses like cadmium (Cd) and lead (Pb). The present study aimed to evaluate the role of Si in chilli plants grown under different concentrations of Cd and Pb respectively. Based on our findings, the increased levels of Cd and Pb adversely affected the physiological and biochemical traits in chilly plants. For instance, at 100 mg kg^−1^, Cd and Pb significantly reduced the seed germination (62.5% and 50%), vigor indices (67.2% and 56.8%), root biomass (88% and 66%), chlorophyll a (75.5% and 55.5%) and carotenoids (56.4% and 48.7%) in chilly plants. However, supplementation of Si in chilli plants aids them in recovering from Cd and Pb side effects by improving their physiological and biochemical traits. At 25 mg kg^−1^ soil Cd and Pb, Si significantly (*p ≤* 0.05) improved the root length (17% and 24%), root biomass (23% and 27%), and carotenoids (16% and 19%) of chilli plants compared to control plants. Moreover, Si application significantly (*p ≤* 0.05) reduced the oxidative stress markers; malondialdehyde (MDA), electrolyte leakage (EL), hydrogen peroxide (H_2_O_2_), and superoxide radical (O_2_-) in Cd and Pb stressed chilli plants as compared to non-Si treated plants. Interestingly, Si foliar application in Cd and Pb-treated chilli plants upregulates the transcript levels of *POD*, *SOD*, *CAT*, and *GPX* genes. Additionally, Si reduces the HM-induced phytotoxicity by decreasing Cd and Pb uptake in roots and shoots of chilli plants, as well as metal translocation (TF) and bioconcentration (BCF) factors. In summary, these results highlight the protective role of Si in chilli plants by mitigating the side effects of Cd and Pb stress. Hence, Si fertilizers can be used in sustainable agriculture to mitigate HM toxicity and improve crop productivity.

## 1 Introduction

Soil contaminated with heavy metals (HMs) poses a risk to ecosystems and human health, both through direct contact with the polluted soil and through the consumption of food grown in such contaminated environments (Golia, 2023). Likewise, HMs toxicity in agriculture soil is a major problem affecting crop growth, yield, and quality (Ali et al., 2022). They can also alter beneficial microbiota and soil physico-biochmical properties thereby reducing soil fertility (Naz et al., 2022). They are resistant to degradation, and if they are not absorbed by plants or eliminated through leaching, they can build up in the soil and remain for extended durations (Rodríguez Eugenio et al., 2018). Currently, anthropogenic activities such as mining, smelting, plastic manufacturing, e-waste processing, excessive use of pesticides and fertilizers, and irrigation with wastewater have significantly increased, leading to elevated levels of harmful chemicals in agricultural soils (Adepoju et al., 2024). The majority of these hazardous contaminants include lead (Pb), cadmium (Cd), chromium (Cr), mercury (Hg), copper (Cu), antimony (Sb), and arsenic (As) (Ali et al., 2021). Plants that absorb these pollutants provide major health risks to people who live in contaminated areas, mostly through the food chain (Uddin et al., 2021). Unlike other organic contaminants, HM pollution in soils can be permanent and last for a very long time (Rajendran et al., 2022). HM contamination poses major risks to plants through soil-to-plant transfer at a faster rate than other organic contaminants (Jalali and Meyari, 2022). The HMs in the soil limit plants’ ability to absorb and translocate vital nutrients, which harms crop development, photosynthesis, biomass, and productivity (Hu et al., 2023). Furthermore, leafy vegetables are more likely to be harmed since they can absorb and store a greater quantity of HMs in their aerial regions (Tauqeer et al., 2022). The large leaf surface area and metabolic activity make them efficient at absorbing and accumulating these metals in their aerial parts (leaves and stems) (Adhikari and Struwig, 2024). Their stomata can absorb pollutants from the air, and metals taken up by the roots are transported to the leaves. This accumulation in the edible parts increases the risk of contamination, especially in polluted environments, and can lead to harmful health effects when consumed (Guo et al., 2023). The pollution caused by heavy metals in the supply chain of food, on the other hand, is made worse by peri-urban agriculture, which exists as a result of the growing need for vegetables and grains. Many studies have shown that HM exposure in humans through food leads to severe diseases such as stomach cancer (Fei et al., 2018). Thus, it is essential to decrease HM uptake and translocation in plants and prevent HM pollution using effective techniques to guarantee safe human health and wholesome food production (Tauqeer et al., 2022).

Fresh vegetables are essential components of every human diet since they are affordable and high in protein, vitamins, minerals, and fiber. Members of the solanaceae family, such as chilli (*Capsicum annuum* L.), are used in several applications, such as food flavoring, natural plant coloring, and medicine (Swamy and Kumar, 2024). The genus Capsicum contains a group of closely related alkaloids called capsaicinoids, which are responsible for the hot flavor of chilli. It also contains retinol, tocopherol, and ascorbic acid. It has been observed to exhibit strong antioxidant activity and anti-mutagenic properties. There have also been reports of its preventive properties against obesity and excessive cholesterol. The chilli crop frequently experiences several abiotic stressors, such as HMs, which result in significant production losses. Therefore, there is a need to develop sustainable practices to improve stress resilience in capsicum crops to meet its high nutritional and global demand. In this study, we examined the role of Si in mitigating the harmful effects of Cd and Pb in chilly plants.

Silicon (Si) is an essential component of the earth’s crust and plays a multifaceted role in terms of growth promotion and stress resilience (Hoffmann et al., 2020; Gulzar et al., 2021). Several studies have demonstrated that Si reduces the harmful effects of HMs on plants cultivated in contaminated soils (Okeke et al., 2023). Previous studies have shown that applying Si to HM-exposed crop plants can have a positive impact on their growth and adaptation (Vaculík et al., 2020). Additionally, Si changes soil phosphorus (P) content, and improves P availability and absorption, which is subsequently absorbed by plant roots together with other vital nutrients (Etesami and Schaller, 2023). The majority of the Si received by plants is stored in or between the cell walls. Several plant species, including *Zea mays* (Rahman et al., 2023), *Oryza sativa* (Peng et al., 2024; Ghouri et al., 2024), *Brassica napus* (Huang et al., 2024), *Brassica rapa* subsp*. pekinensis* (Fu et al., 2023), *Vigna radiata* (Thakral et al., 2024), *Triticum aestivum* (Alshegaihi et al., 2024) showed a notable improvement in their growth under HM stress when Si was present. Generally Si reduces the intake of HM by chelating or arresting them in the soil, which also reduced their bioavailability and may have limited their movement from roots to shoots (Thakur et al., 2023). The HM and Si co-precipitation contributes to cell wall thickness by developing robust silica barriers that bind and prevent the transport of metals (Khan et al., 2021). Si or Si-based fertilizers added to the soil can change the physiochemical characteristics of the soil, the structure of the bacterial population, and the transformation, immobilization, and limitation of metal contaminants (Rajput et al., 2021). In plants, Si-based defense mechanisms include: (1) increase in ROS detoxification by activating the antioxidant defense system; (2) repairing damaged cell membranes; (3) improving the photosynthetic machinery; (4) enhancing the uptake/accumulation of essential nutrients; (5) moving HMs to the subcellular level; and (6) removing HMs from the cells (Rachappanavar et al., 2024). Si serves a multitude of biological purposes in plants, including morphological changes, and improvements in photosynthetic capacity through an increase in grana, chloroplast size, and chlorophyll concentrations, which ultimately increase the dry biomass (Bu et al., 2024). In addition, Si creates a cuticle-double Si layer in the leaves that lessens water transpiration and disease and pest attacks (Pereira et al., 2024).

The present study aimed to evaluate the role of Si in alleviating the toxicity of Cd and Pb in chili plants. We conducted a thorough investigation into how Si influences physiological and biochemical characteristics related to growth and stress tolerance in chili plants under Cd and Pb stress.

## 2 Materials and methods

### 2.1 Seed germination evaluation under HM-stress

To evaluate the impact of silicon (Si) alone or in combination with heavy metals (Cd and Pb), in chilli plants seeds (var. Kashi Anmol) were obtained from the Indian Institute of Vegetable Research (IIVR), Varanasi, Uttar Pradesh, India. The seeds were immersed in double-distilled deionized water (DDW) for 24 h. After washing, seeds were surface sterilized with 1% sodium hypochlorite (NaOCl) for 5 min. and were rinsed three times with sterile DDW. Soft agar plates (0.7%) containing varying concentrations (0, 25, 50, and 100 mg kg^-1^) of lead (Pb) and cadmium (Cd) were then prepared. Eight seeds per plate were subsequently placed on the soft agar plates and incubated at room temperature for three to four days. For each treatment, five plates were used, and the experiments were conducted in triplicates. After four days, measurements of the root and shoot lengths of the plantlets, along with the germination percentage, were recorded (Altaf et al., 2024a).

### 2.2 Evaluating the effect of Si on Cd and Pb-stressed chilli plants: pot experiments

#### 2.2.1 Chilli seeds, HMs treatment, and Si application

The chilli seeds that were healthy and uniformly sized were sterilized for five minutes using 1% sodium hypochlorite (NaOCl) and then were rinsed with double distilled water (DDW). The chemicals used in this study were acquired from Sigma-Aldrich. The sodium silicate (Na_2_SiO_3_; 1.5 mM) was applied as a source of silicon (Si) to metal-stressed chilli seedlings. The requisite amounts of cadmium chloride (CdCl_2_) and lead acetate (Pb [CH_3_COO]_2_) salts were made by dissolving them in deionized water, with the final volume being kept at 100 mL in a volumetric flask. The 25, 50, and 100 mg kg^-1^ concentrations of both HMs (i.e. Cd and Pb) were used throughout the experiment.

#### 2.2.2 Experimental design

The Si-untreated and treated seeds (n= 8) of chilli (*Capsicum annuum* L.) were sown in plastic pots containing 1.0 kg of unsterilized soils (sandy clay loam and had organic C 5.88 g kg^−1^, Kjeldahl N 0.943 g kg^−1^, Olsen P 19.7 mg kg^−1^, pH 7.0 and WHC 0.39 ml g^−1^, cation exchange capacity 8.54 and 3.77 cmol kg^−1^ anion exchange capacity) treated with 0, 25, 50 and 100 mg kg^−1^ soils of Cd and Pb. The heavy metal solution was prepared and each experimental pot was supplemented individually with varying HM solutions. As a source of silicon (Si), sodium silicate (Na_2_SiO_3_) was used because it is readily available and widely used source of soluble Si for plant experiments. A concentration of 1.5 mM Si (Na_2_SiO_3_) was used in this experiment, as several studies have shown that 1–2 mM Na_2_SiO_3_ enhances stress tolerance without causing phytotoxicity. And 1.5 mM concentration falls in optimal range (effective but not excessive). Higher concentrations (>2.0 mM) could cause ionic imbalance or alkalinity stress (Hou et al., 2023; Huang et al., 2018; Abdel Latef and Tran, 2016; Kim et al., 2014). The Si foliar application to soil-grown chili plants was carried out at the early vegetative stage (approx. 4 weeks after sowing) and again at pre-flowering stage. Silicon was applied two times during the growth cycle. There was a total of 14 treatments for HMs treatments including untreated control and a total of 39 earthen pots. The treatment plans were as follows: T1 = control (C), T2 = Silicon (Si) treatment only, T3 = 25 mgCdkg^−1^ soil, T4 = 50 mgCdkg^−1^ soil, T5 = 100 mgCdkg^−1^ soil, T6 = 25 mgPbkg^−1^ soil, T7 = 50 mgPbkg^−1^ soil, T8 = 100 mgPbkg^−1^ soil, T9 = 25 mgCdkg^−1^ soil + 1.5 mM Si, T10 = 50 mgCdkg^−1^ soil + 1.5 mM Si, T11 = 100 mgCdkg^−1^ soil + 1.5 mM Si, T12 = 25 mgPbkg^−1^ soil + 1.5 mM Si, T13 = 50 mgPbkg^−1^ soil + 1.5 mM Si, T14 = 100 mgPbkg^−1^ soil + 1.5 mM Si. Each treatment was performed in triplicate (*n* = 3), and all pots were arranged in a block configuration that was entirely randomized. Pots were kept in an open field condition (D/N:25–30°C/18–22°C; 12–14 h light/dark, relative humidity 60-80%; pH of 6–7) between March to May and watered as required. Thinning was performed 15 days after emergence (DAE), and three plants were kept in each pot and were further used for the treatments.

### 2.3 Growth and biomass determination

Twenty days after sowing (DAS), chilli plants from each treatment were collected and separated into two sections: above-ground and below-ground part. Fresh biomass was estimated for both above- and below-ground plant components. After four days of drying shoots and roots at 60°C, the dry biomass of above- and below-ground components was calculated using a conventional weight balance (Shafi et al., 2024; Shahid et al., 2024).

### 2.4 Analyses of carotenoid and chlorophyll contents

To estimate the leaf pigments in leaf tissues of HM-treated and Si-applied chili plants, approximately 0.5 g of fresh leaf tissues were cut and immersed in 80% cooled acetone for 24 h. Finally, the mixture was centrifuged for 10 min. at 4000 rpm (4°C). The concentrations of chlorophyll a, b, total chlorophyll, and carotenoids, in the supernatant, were measured spectrophotometrically and readings were recorded at wavelengths of 479 nm, 663 nm, and 645 nm, respectively (Arnon, 1949; Kirk and Allen, 1965).

### 2.5 Antioxidant enzyme activity determination in HMs stressed and Si-applied chilli plants

To extract the antioxidant enzymes accumulated in leaf tissues of Cd and Pb-treated and Si-applied chilli plant, 0.5 g of fresh leaf tissue was homogenized using a cooled pestle and mortar in 50 mM, pH 7.0 cold phosphate buffer that was supplemented with 1% polyvinyl pyrrolidine (PVP) and 1 mM EDTA. After centrifuging at 15,000 × *g* for 20 min. at 4°C, the supernatant was collected and utilized as a source of enzymes. The amount of protein in the supernatant was determined using Bradford (1976). The activity of superoxide dismutase (SOD, EC 1.15.1.1) was measured using the procedure described (Romanowska et al., 2008). An assay combination including sodium phosphate buffer (50 mM, pH 7.5), 100 µL of EDTA, L-methionine (13 mM), NBT (75 µM), riboflavin (75 µM), and 100 µL of enzyme extract was used to record the inhibition of photochemical reduction of nitro blue tetrazolium chloride (NBT) at 560 nm. After 15 min. of illumination, the process was stopped by turning off the light, and the absorbance at 560 nm was measured in comparison to the blank that was not lighted. The EU mg^-1^ protein was used to express the activity of SOD, which was defined as an amount of enzyme that caused 50% inhibition in the photoreduction of NBT. The activity of catalase (CAT, EC1.11.1.6) was measured by tracking the change in optical density at 240 nm for two min. The reaction mixture included 100 µl of enzyme, 0.1 mM EDTA, hydrogen peroxide (H_2_O_2_), and phosphate buffer (100 mM; pH 7.0). Utilizing an extinction value of 39.4 mM^−1^ cm^−1^, the activity was represented as U mg^−1^ protein (López et al., 2007).

### 2.6 Gene expression analysis of HM-treated and Si-applied chillis

Total RNA was extracted from the leaf samples of Cd and Pb-stressed, and Si-applied chilli plants using a Chromous RNA isolation kit. For cDNA synthesis, a total of 2.0 μg of RNA, 10 mM deoxynucleotide triphosphates (dNTPs), and water were utilized following the manufacturer’s guidelines. After being further diluted, the cDNA was employed for real-time (RT) PCR. The 2.0 μl of first strand cDNA in a 20 μl reaction with 10 μl of iQ-SYBR Green Supermix (Bio-Rad, USA), 0.8 μl of 100ng/μl of each gene-specific primer pair, and 6.4 μl of Milli-Q water. Using qRT-PCR, changes in the gene expression patterns of defense-related genes *CAT*, *GPX*, *POD*, and *SOD* were identified (**Supplementary Table 1**). A single cycle of qRT-PCR was conducted at 94°C (5 min), followed by 35 cycles at 95°C (20 sec), annealing according to each primer’s Tm (1 min), and extension at 72°C (30 sec). Cycle threshold values (CT) were calculated by analyzing the output from the real-time software. The ΔΔCT technique was used to calculate the relative expression.

### 2.7 Oxidative stress biomarkers of HM-stressed and Si-treated chilli plants

#### 2.7.1 Determination of free proline

Bates et al. (1973) procedure were utilized to estimate the proline content in Cd and Pb-treated chilli plants in response to Si application. The plant material was homogenized with 3% aqueous sulfosalicylic acid and then centrifuged at 15,000 rpm. The supernatant was used to assess the concentration of proline. The reaction mixture, consisting of 2.0 ml of acid ninhydrin and 2.0 ml of glacial acetic acid, was heated at 100°C for one hour. After the reaction was completed in an ice bath, 4 ml of toluene was used to extract the reaction mixture, and the absorbance at 520 nm was measured.

#### 2.7.2 Determination of malondialdehyde

The MDA content in leaf tissues of Cd/Pb and Si-treated chilly plants was measured using the method described by Heath and Packer (1968). After extracting 0.2 g of fresh leaf in 5 ml of trichloroacetic acid (TCA; 0.1% w/v), the extract was centrifuged at 12,000 × *g* for 5 min. at 4°C. After adding 20% TCA to the extracted solution and mixing it with 4 ml of 0.5% thiobarbituric acid (TBA), the mixture was placed in a hot water bath set at 90°C for 30 min. The final extract was placed on ice. The absorbance values were measured at 532 nm.

#### 2.7.3 Electrolyte leakage measurement

Electrolyte leakage was measured using Dionisio-Sese and Tobita (1998) method. Twenty leaf discs were taken in a boiling tube having 10 ml of deionized water. The contents were heated in a water bath for 25 min. at 50 and 60°C, respectively, and the EC was measured (EC_b_). After ten minutes of boiling at 100°C, the contents’ EC was measured again (EC_c_).

### 2.8 Determination of reactive oxygen species

#### 2.8.1 Hydrogen peroxide and superoxide content measurements

The effect of Si on H_2_O_2_ content in Cd and Pb-stressed chilli plants was determined (Shahid and Singh, 2024a, b). For the assay, 4 ml of 5% trichloroacetic acid (TCA) was used to homogenize the 0.2 g of frozen roots from each pot. After centrifugation at 10,000 rpm for 15 min. at 4°C, 0.5 ml of the supernatant was combined with 0.5 ml of phosphate buffer (0.1 M, pH = 7.0) and 1.0 ml of KI (1 M), and the mixture was incubated for 1 h in the dark. At 390 nm, the mixture’s absorbance was measured, and a standard curve was used to calculate the H_2_O_2_ concentration.

### 2.9 Measurements of Pb concentration in chilli

To determine the Pb content in the roots and shoots of chilli plants, the samples were washed with double distilled water and dried at 60°C until their weight remained constant. A Teflon bottle holding 0.2 g of plant samples was filled with nitric acid/perchloric acid (HNO_3_-HClO_4_; V: V=6:2) and kept on a hotplate at 180°C until 1.0 ml of liquid remained in the tube. Following sample dilution, the final quantity of lead in the solution was estimated using an Agilent 7700 inductively coupled plasma mass spectrometry (ICP-MS) (Agilent Technologies, CA, USA).

### 2.10 Cd concentration determination and metal translocation factor calculation

The Cd content was measured using Huang et al. (2021) with minor modifications. The root and shoot samples were dried and digested on heated hot plates with a combination of nitric acid (HNO_3_) and perchloric acid (HClO_4_) acid until a clear digestion liquid was obtained. To prepare for measurement, the cooled digestion liquid was diluted to 50 ml with 1% (v/v) nitric acid and filtered. Cd concentration in each sample was evaluated using an atomic absorption spectrometer (AAS; Model AA-6800, Shimadzu Corporation, Japan). The translocation factor was evaluated to determine the phytoremediation ability of Pb and Cd-treated and Si-applied chilli plants (Pachura et al., 2016).

### 2.11 Statistical analysis

Every experiment was conducted in three repetitions (*n* = 3) of every treatment, with three plants in each pot. Mean values are displayed along with their corresponding standard deviation (mean ± S. D.). One-way analysis of variance (ANOVA) and Duncan’s multiple range test (DMRT) were performed using SPSS 21.0 software (SPSS, Inc., Chicago, IL, USA) to determine the significant difference between treatments at 5% level (*p*< 0.05). To examine the relationship between the examined parameters, Origin Pro 2018 and ClustVis tools were used to conduct principal components analysis (PCA), person correlation matrix, and heat map studies.

## 3 Results

### 3.1 Effect of exogenously applied Si on germination efficiency and vigor index under HM stress

**Figure 1:**
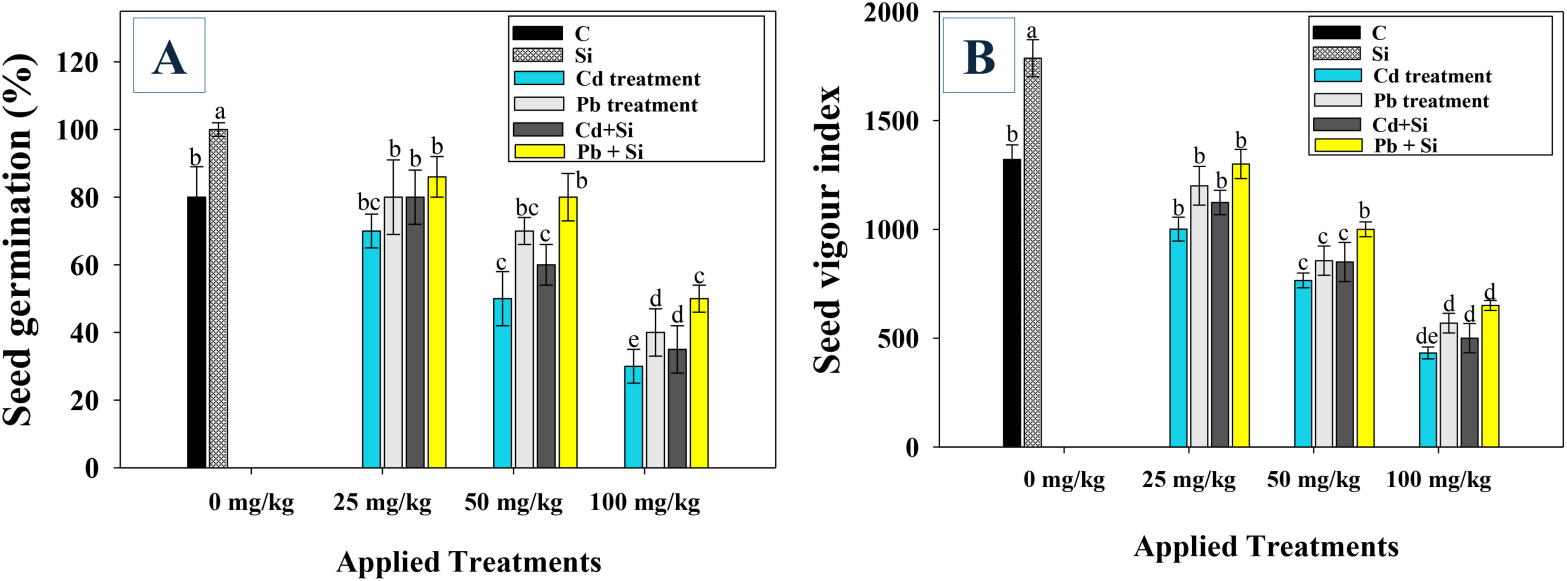
Effect of exogenously applied silicon (Si) on germination efficiency **(A)**, and seedling vigour index **(B)** in chilli plants treated with Cd and Pb. Here, C, control; Si, silicon; Cd, cadmium; Pb, lead. The bar diagrams represent the mean values of three replicates (*n* = 3). Corresponding error bars represents standard deviation (S.D.). Letters a, b, c, d etc. in figure depict that mean values are significantly different from each other according to Duncan’s multiple range test (DMRT).

The Cd and Pb adversely affected the seedling germination efficiency and vigor index (SVI) in chilli plants. Compared to lower doses, higher concentrations caused the maximum reduction in germination attributes. For example, Cd and Pb at 100 mg kg^−1^ soil, significantly (*p ≤* 0.05) decreased the germination efficiency by 62.5% and 50%, and SVI by 67.2% and 56.8%, respectively (**Figure 1A**). However, exogenously applied Si reversed the toxicity and improved the chilli germination ability. When applied, 25 and 50 mg kg^−1^ soil Cd and Pb stressed chillis, Si increased the germination efficiency by 16.6, 14%, and 25, 17%, respectively, over non-Si treated chilli plants (**Figure 1B**). Similarly, Si application significantly (*p ≤* 0.05) increased the vigor indices by 23 and 29.4% when foliarly applied to 25 mg kg^-1^ soil treated Cd and Pb, respectively, over non-Si treated chilli plants.

### 3.2 Si improves vegetative growth and biomass of Cd and Pb-exposed chillis

**Figure 2:**
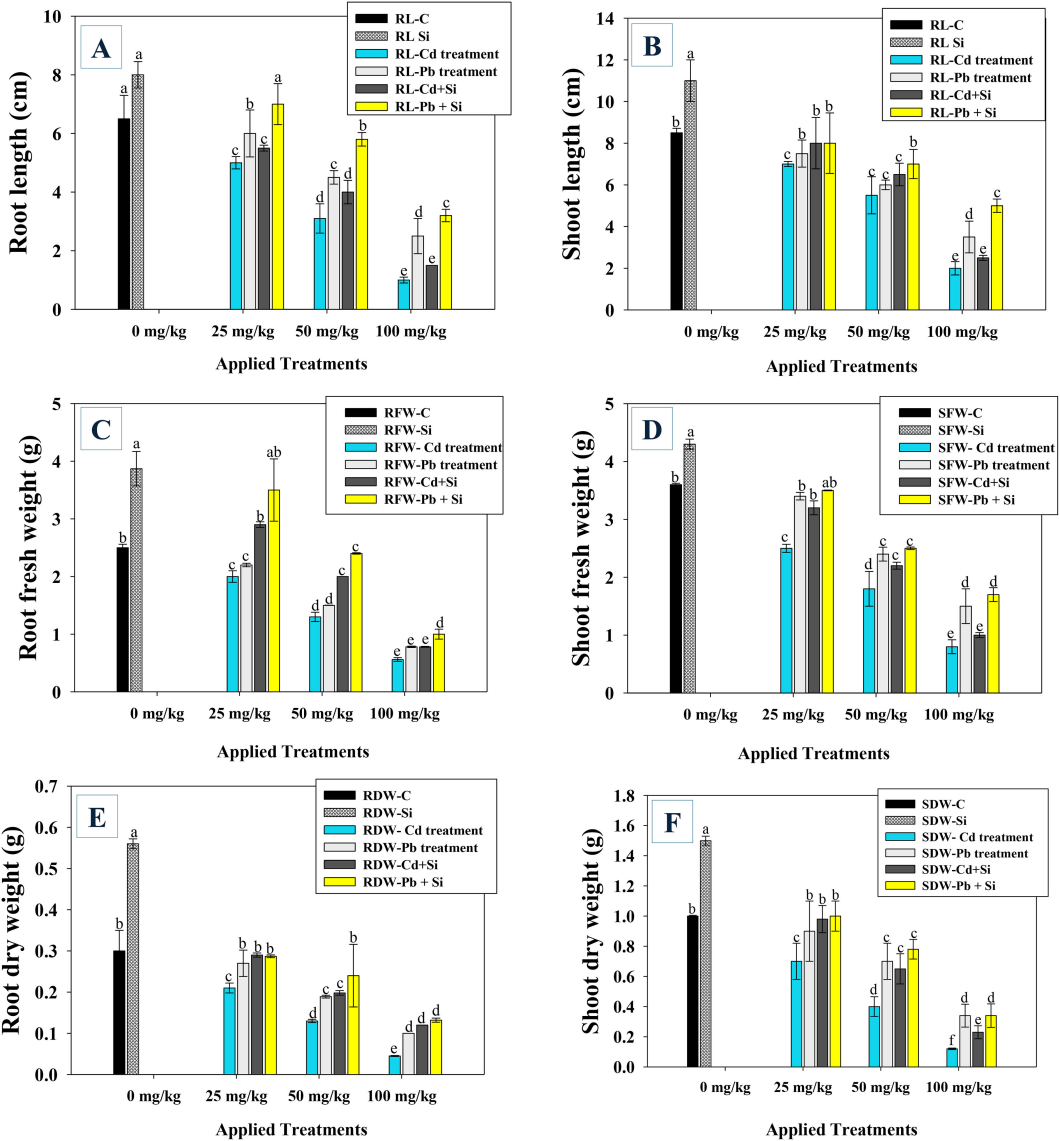
Effect of silicon (Si) root length **(A)**, shoot length **(B)**, root fresh weight **(C)**, shoot fresh weight **(D)**, root dry weight **(E)** and shoot dry weight **(F)** of chilli plants treated with different Cd and Pb concentrations. Here, C, control; Si, silicon; RL, root length; SL, shoot length; RFW, root fresh weight; SFW, shoot fresh weight; RDW, root dry weight; SDW, shoot dry weight; Cd, cadmium; Pb, lead. Each experiment was independently repeated thrice. The bar and scatter diagrams represent the mean values of three replicates (*n* = 3), with sample size (n =5). Corresponding error bars represents standard deviation (S.D.). Letters a, b, c, d etc. in figure depict that mean values are significantly different from each other according to DMRT test.

The chilli plants cultivated in soil treated with Cd and Pb and applied with Si showed varying growth characteristics (**Supplementary Figure S1**) and biomass. As the dosage of Cd and Pb increased, the fresh weights of roots (RFW), shoots (SFW), lengths of roots (RL), and shoots (SL) significantly (*p* ≤ 0.05) decreased in both the treatments. However, Si application caused the metal reduction and improved the growth of chilli seedlings (**Figures 2A–F**). The Cd and Pb at 100 mg kg^−1^ soil, for example, significantly (*p* ≤ 0.05) decreased the RL (67.4 and 48.5%), SL (45.6 and 39.2%) (**Figures 2A, B**), RFW (73.4 and 52.3%), and SFW (55.7 and 36.9%) (**Figures 2C, D**). Similarly, the dry biomass of chilli was adversely affected by metal exposure. On the other hand, even under metal pressure, adding silicon (Si) to metal-stressed plants enhanced their growth and biomass characteristics and reduced their toxicity. For instance, under 25 mg kg^−1^ soil Cd and Pb, the administration of Si enhanced the lengths of roots (33.5 and 51.5%), shoots (33.4 and 45%) and biomass of roots (38.7 and 51%), and shoots (36.5 and 39.7%) compared to the identical metal treatment (**Figures 2E, F**).

### 3.3 Si improved the leaf pigments in metal stressed chilli plants

**Figure 3:**
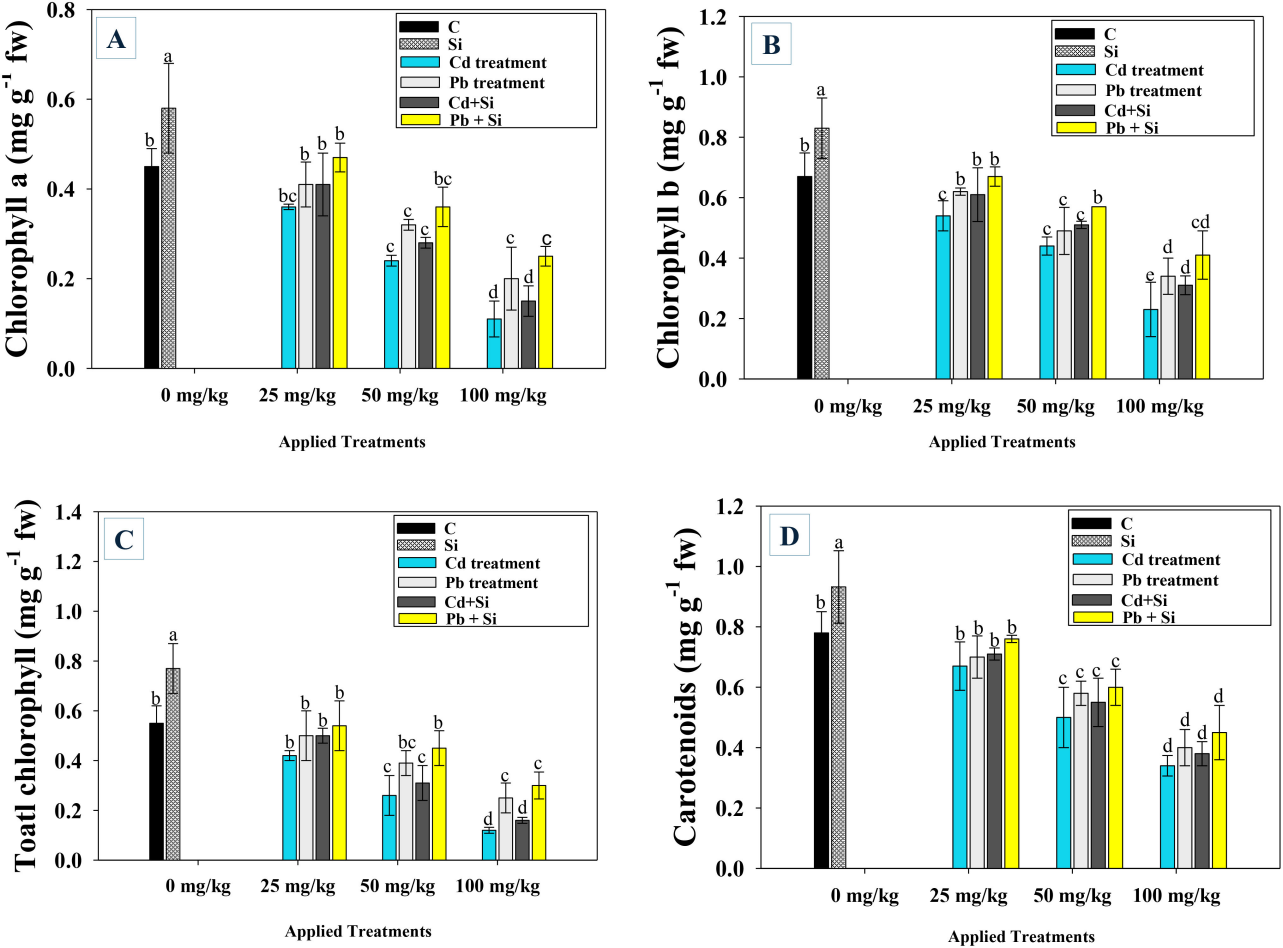
Impact of Si on leaf pigments; chlorophyll a **(A)**, chlorophyll b **(B)** total chlorophyll **(C)** and carotenoid content **(D)** of chilli plants exposed to varying Cd and Pb concentrations. Each experiment was independently repeated thrice. The bar and scatter diagrams represent the mean values of three replicates (*n* = 3), with sample size (n =5). Corresponding error bars represents standard deviation (S.D.). Letters a, b, c, d etc. in figure depict that mean values are significantly different from each other according to DMRT test.

Like growth characteristics, Cd and Pb had a detrimental impact on the accumulation of leaf pigments (carotenoids and chlorophyll) in chilli plants treated/applied with metal and Si, respectively. Higher metal dosages produced more harmful effects and diminished leaf pigments than lower ones. In comparison to untreated plants, the levels of chlorophyll a (**Figure 3**), chlorophyll b (**Figure 3**), total chlorophyll (**Figure 3**), and carotenoids (**Figure 3**) were maximally and significantly decreased by 75.5, 56.7, 63.2 and 48.3%, respectively, at 100 mg Cd kg^−1^. The application of Si reduced metal toxicity and improved the accumulation of pigments even under metal stress. For instance, when applied in the presence of 25 mg kg^−1^ Cd and Pb, Si improved chlorophyll a (20 and 25%), chlorophyll b (14 and 11%), total chlorophyll (23 and 31%), and carotenoids (26.4 and 33.0%) over metal stressed chilli plants.

### 3.4 Si modulated the antioxidant enzyme system of metal-stressed chilli plants

**Figure 4:**
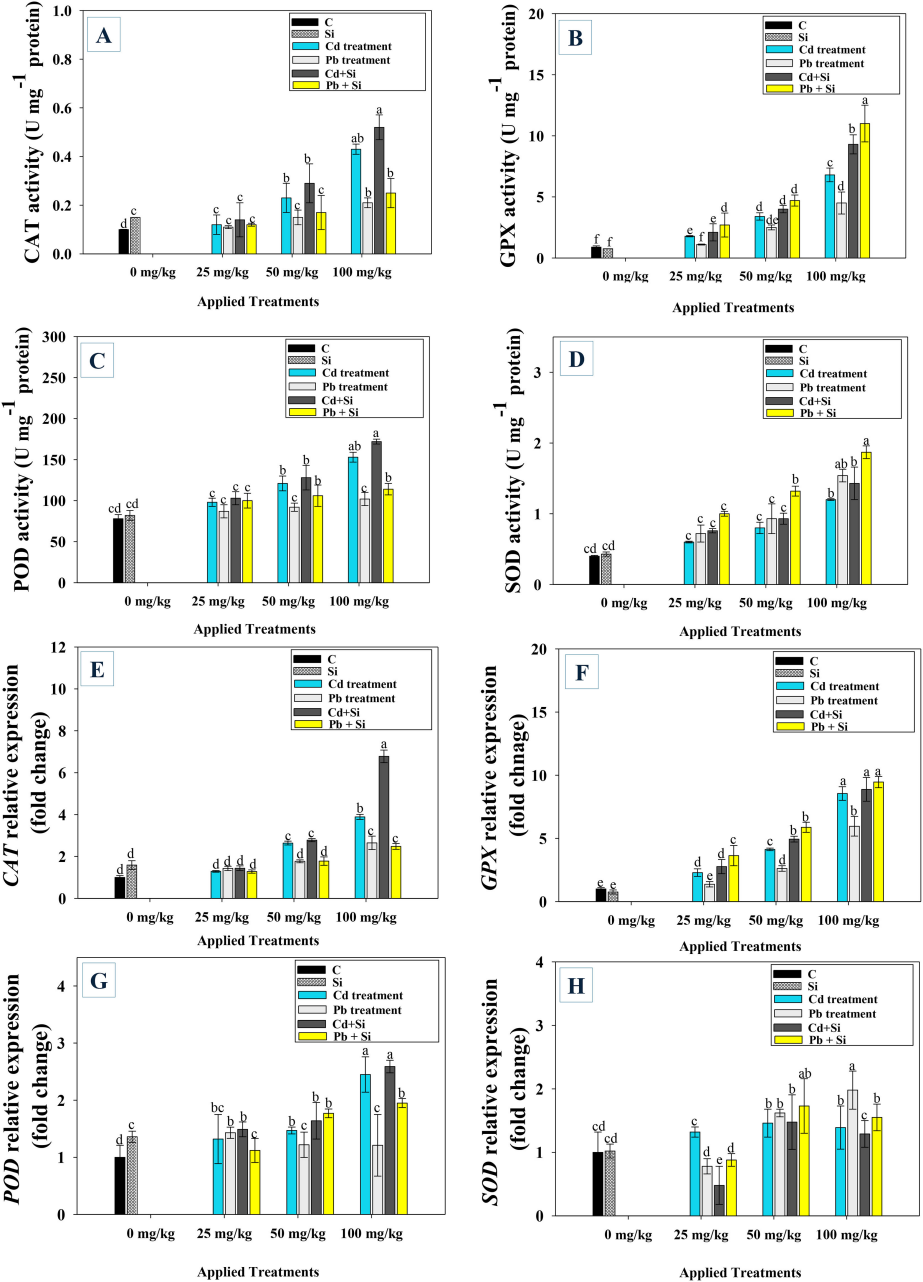
Antioxidant enzymes; catalase **(A)**, guaiacol peroxidase **(B)**, peroxidase **(C)**, and superoxide dismutase **(D)** and defense responsive gene expressions i.e. *CAT* **(E)**, *GPX* **(F)**, *POD* **(G)** and *SOD* **(H)** of chilli plants treated with Si and increasing Cd and Pb concentrations. Each experiment was independently repeated thrice. The bar and scatter diagrams represent the mean values of three replicates (*n* = 3), with sample size (n =5). Corresponding error bars represents standard deviation (S.D.). Letters a, b, c, d etc. in figure depict that mean values are significantly different from each other according to DMRT test.

The levels of antioxidant enzymes were increased in a concentration-dependent way. Under control conditions, the activities of CAT (**Figure 4**), GPX (**Figure 4**), POD (**Figure 4**) and SOD (**Figure 4**) remained unchanged, although marginally increased with lower Cd and Pb doses. Higher metal dosages, however, markedly raised the activity of the enzyme. For example, at 100 mg kg^−1^ soil, Cd and Pb increased the activity of CAT (54% and 79%), GPX (63% and 55%), POD (39% and 61%) and SOD (41% and 51%) compared to the untreated control. But under metal stress, foliar spraying with Si enhanced enzyme activity in comparison to their respective controls. For instance, in the presence of 25 mg kg^−1^ soil Cd and Pb significantly (*p* ≤ 0.05) increased the activities of CAT (23% and 29%), GPX (43% and 54%), POD (29% and 53%) and SOD (48 and 37%) compared to non Si treated plants.

### 3.5 Si regulates the expression levels of antioxidant defense responsive genes

In this study we systematically assessed the expression levels of antioxidant genes in chilli plants after HM stress and Si supplementation. Silicon alone increased the *CAT* expression by 1.59-fold. Cadmium treatments showed a progressive increase in *CAT* expression, with the highest value (3.79-fold) compared to control under 100 mg kg^−1^. The Pb treatments also increased the CAT expression, with the highest (2.65-fold) observed at 100 mg kg^−1^ compared to the control. Combining Si with Cd resulted in a significant increase, especially at 100 mg kg^−1^ of Cd (6.78-fold), higher compared to the control. Silicon combined with lead also elevated the CAT expression, but the effect was less pronounced compared to the Cd treatments. The highest *CAT* expression was observed with Si+ Cd 100 mgkg^−1^ (**Figure 4**). Si treatment increased the *POD* gene expression by 1.36-fold compared to the control. Cd treatments showed a progressive increase in *POD* gene expression, with the highest value (2.45-fold), higher compared to the control observed at 100 mg kg^−1^ concentration. Pb treatments generally resulted in lower *POD* gene expression, with the value lowest at 100 mg kg^−1^ concentration (1.21-fold), compared to the control. Combining Si with Cd led to a notable increase, especially at 100 mg kg^−1^ of Cd, where the expression level reached 2.59-fold higher. Si with Pb showed an enhancement in *POD* gene expression, with the highest value at 100 mg Pb kg^−1^ (1.95-fold) (**Figure 4**). The combination of Si and Cd resulted in the most significant increase in *POD* gene expression. Si treatment increased the *POD* gene expression by 1.36-fold and the *SOD* gene expression by 1.02-fold. The Cd treatments showed a gradual increase in the *POD* gene expression, with the highest expression of 2.45-fold under 100 mg kg^−1^ treatments, while the *SOD* expression peaked by 1.46-fold under Cd 50 mg kg^−1^ treatments. Pb treatments generally resulted in lower *POD* expression, with the lowest expression of 1.21-fold under 100 mg kg^−1^ concentration, and the *SOD* expression varied, with the highest (1.98-fold), compared to the control, under 100 mg kg^−1^ Pb concentration. Combining Si with Cd resulted in higher *POD* gene expression, particularly at 100 mg Cd kg^−1^, where it enhanced by 2.59-fold compared to the control (**Figure 4**). The Si + Cd treatments had variable effects on SOD, with the highest expression increase of 1.47-fold at 50 mg kg^−1^ concentration of Cd. Si combined with Pb also increased *POD* gene expression, with a 1.95-fold increase at 100 mg kg^−1^ of Pb, and the SOD expression ranged from 0.88-fold under 25 mg kg^−1^ Pb to 1.73-fold under 50 mg/kg Pb (**Figure 4**).

### 3.6 Si detoxified the metal-induced oxidative stress in chilli plants

#### 3.6.1 Proline and malondialdehyde

**Figure 5:**
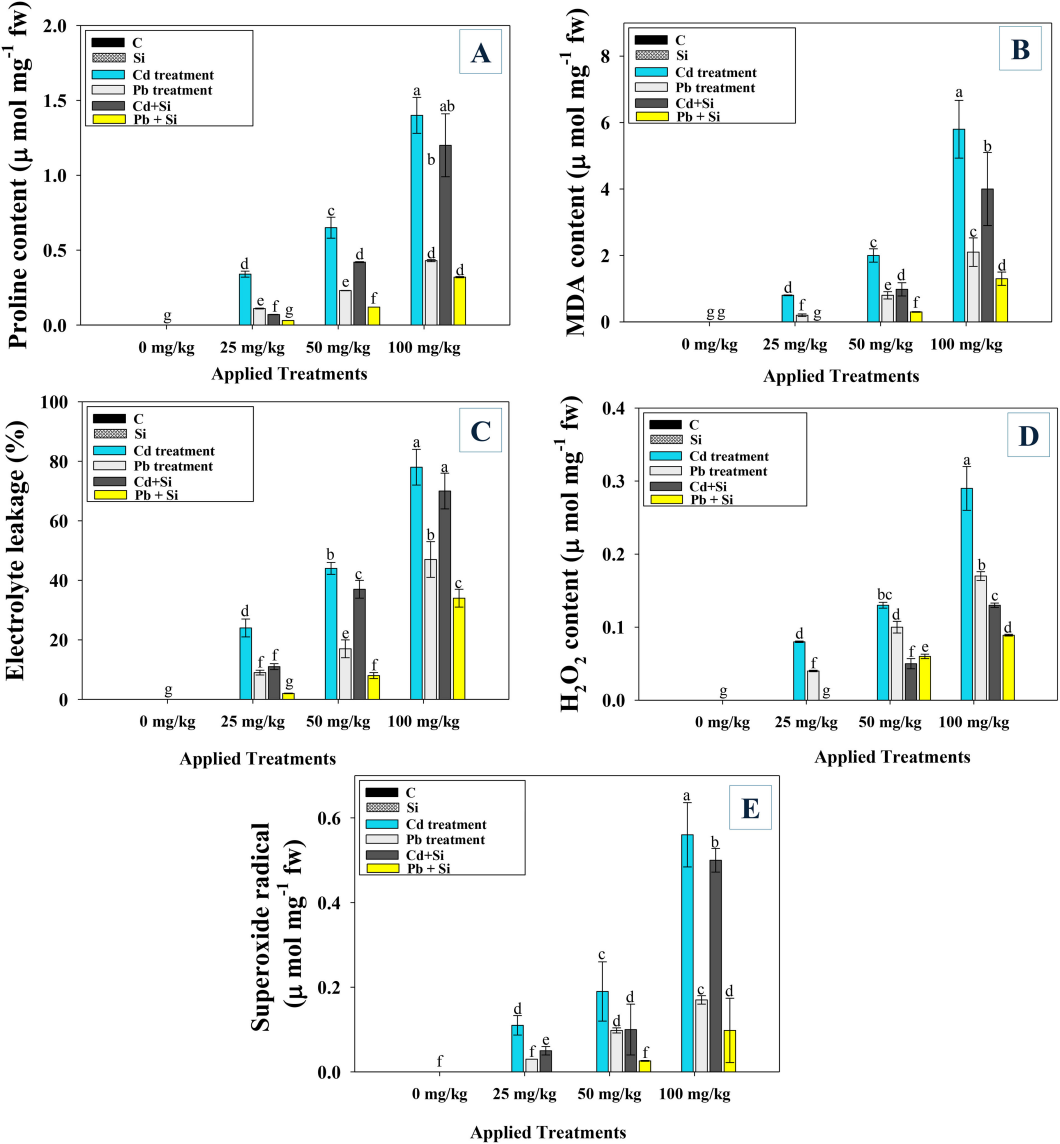
Effect of Si application on proline **(A)**, MDA **(B)**, electrolyte leakage **(C)** and ROS generation; hydrogen peroxide **(D)** and superoxide radical **(E)** content of CD and Pb treated chilli plants. Each experiment was independently repeated thrice. The bar and scatter diagrams represent the mean values of three replicates (*n* = 3), with sample size (n =5). Corresponding error bars represents standard deviation (S.D.). Letters a, b, c, d etc. in figure depict that mean values are significantly different from each other according to DMRT test.

The oxidative stress markers i.e. proline and MDA content in Cd and Pb-stressed and Si applied chilli plants significantly varies among treatments. The accumulation of MDA and proline in chili plants increased with increasing metal concentrations. For instance, Cd and Pb at 100 mg kg^−1^ soil, significantly (*p* ≤ 0.05) enhanced the levels of proline (92.9% and 84%) (**Figure 5**) and MDA (87% and 61%) over untreated control (**Figure 5**). In contrast, Si application potentially detoxified the metal toxicity and lowered the extent of oxidative stress biomarkers in chilli plants. For instance, Si reduced the level of proline in 25, 50, and 100 mg Cd kg^−1^ soil-stressed chilli plant by 66%, 49%, and 37%, respectively, over untreated control. Similarly, while assessing the impact of Cd, Pb, and Si on oxidative stress parameters, they reduced the level of MDA in leaf tissues of metal-stressed soil in the order: 25 mg Cd kg^−1^ (55% and 71%) > 50 mg Cd kg^−1^ (32% and 40%) > 100 mg Cd kg^−1^ (17% and 27%).

#### 3.6.2 Electrolyte leakage, hydrogen peroxide, and superoxide radical

To assess the membrane integrity of Cd-Pb stressed and Si-treated chilly plants, the electrolyte leakage (EL) in the leaves was measured. The levels of EL, H_2_O_2_ and superoxide radicals in Si-applied chilli plants were marginally elevated by the lower dosages Cd and Pb. Among both the metals, higher Cd concentrations, however, were associated with greater levels of toxicity and damage, and oxidative stress was considerably (*p* ≤ 0.05) elevated. For instance, Cd at 100 mg kg^−1^ soil increased the EL by 66% (**Figure 5**), H_2_O_2_ by 83% (**Figure 5**), and superoxide by 73% (**Figure 5**), compared to untreated control. On the other hand, exogenously administered Si enhanced plant development and decreased the degree of oxidative stress. For instance, when given to chilly plants grown in soil treated with lesser dosages of Cd and Pb, Si decreased the levels of EL (56 and 70%), H_2_O_2_ (49 and 67%) and superoxide (51 and 68%) over non Si treated plants.

### 3.7 Metal accumulation in chilli plants in response to Si

**Figure 6:**
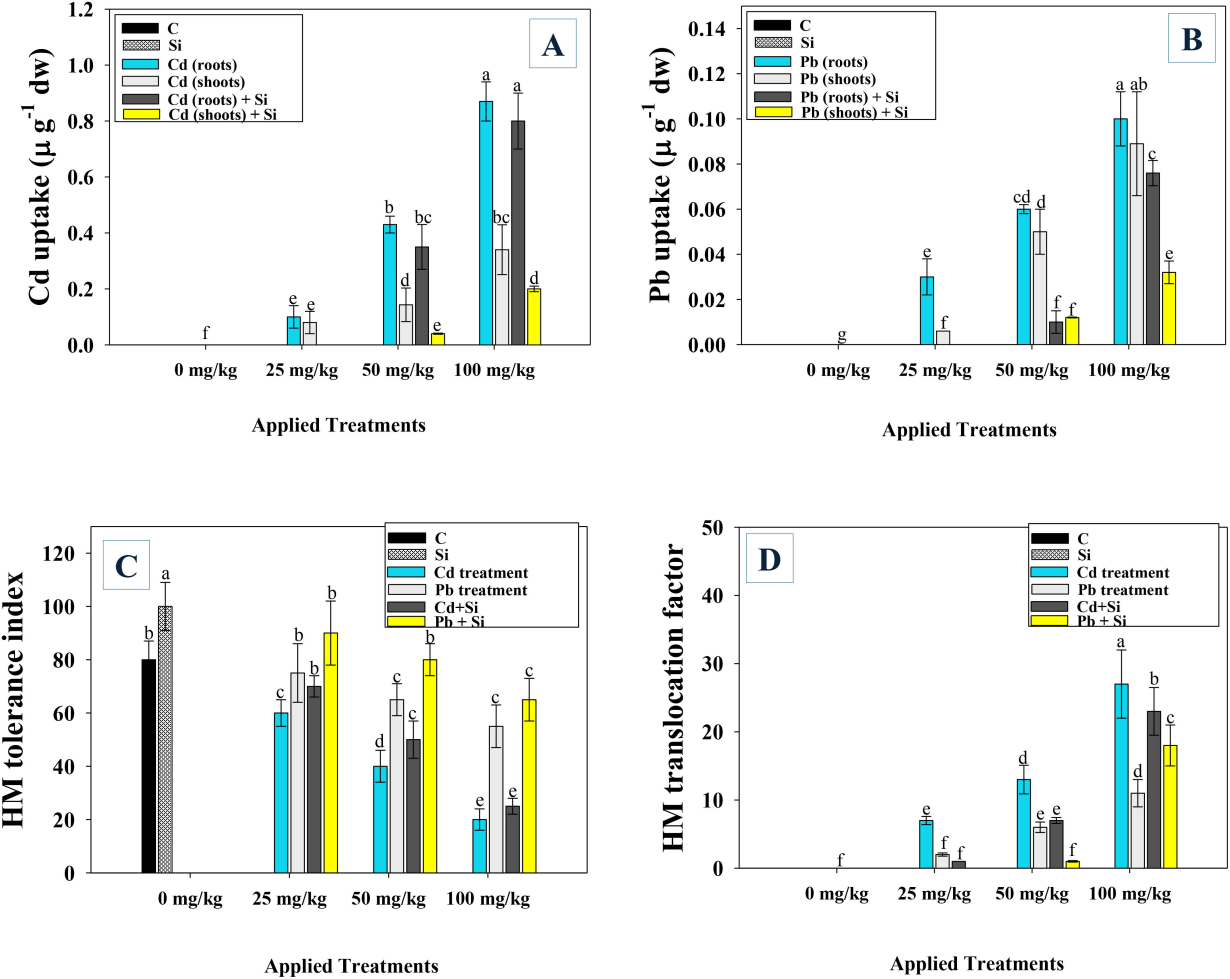
Uptake of Cd **(A)**, Pb **(B)** in roots and shoots tissues, HM tolerance index **(C)** and translocation factor **(D)** of metal-treated and Si applied plants. Each experiment was independently repeated thrice. The bar and scatter diagrams represent the mean values of three replicates (*n* = 3), with sample size (n =5). Corresponding error bars represents standard deviation (S.D.). Letters a, b, c, d etc. in figure depict that mean values are significantly different from each other according to DMRT test.

The Cd and Pb-treated and Si-applied chilli plants exhibited different levels of metal uptake respectively. With increasing metal concentrations, the chilli roots and shoot tissues showed enhanced Cd and Pb uptake. Compared to the shoots, root tissues accumulate the maximum amounts of metals. Less Cd and Pb was absorbed by the plant organs grown in environments with lower metal concentrations. For instance, 0.1, 0.43, and 0.87 µg Cd g^−1^ were detected in root tissues, while 0.08, 0.143, and 0.34 µg Pb g^−1^ in chilly plants were detached from soil contaminated with 25, 50, and 100 mg kg^−1^ of Cd and Pb, respectively. However, the application of Si considerably decreased the uptake of metals in plant organs. As an example, Si treatment significantly (*p*< 0.05) reduced Cd uptake in root tissues by 93.5%, 19%, and 11% and in shoots by 100%, 38%, and 22% when applied to chilli plants added separately with 25, 50, and 100 mg Cd kg^−1^ soil. Similarly, Si application potentially lowered the uptake of Pb in roots (70%) and shoots (100%) of 25 mg kg^−1^ stressed chilli plants (**Figure 6A**).

### 3.8 Effect of Si on metal tolerance index, and translocation factor

Under increasing Cd and Pb concentrations, the TI (**Figure 6**) and TF (**Figure 6**) of chilli plants were decreased and increased, respectively. The Cd at 50 and 100 mg kg^−1^ soil, reduced the TI by 50 and 75%, whereas, at the same concentrations, Pb decreased the tolerance indices of chilli plants by 19 and 32%, respectively, over untreated control. However, when Si was applied to metal stressed plant, the TI of the chilli was improved. For example, compared to plants co-cultivated with 25 and 50 mg Cd kg^−1^ soil, Si increased the tolerance index by 16 and 20%, respectively. Similarly, translocation factors (TF) of metal-stressed chilli plants were increased with increasing Cd and Pb doses. However, the application of Si caused a significant reduction in the TF. For instance, in the presence of 25, 50, and 100 mg Pb kg^−1^ soil, Si supplementation reduced the TF by 39, 28, and 18%, respectively, over Pb treatment only.

### 3.9 Multivariate analysis

**Figure 7:**
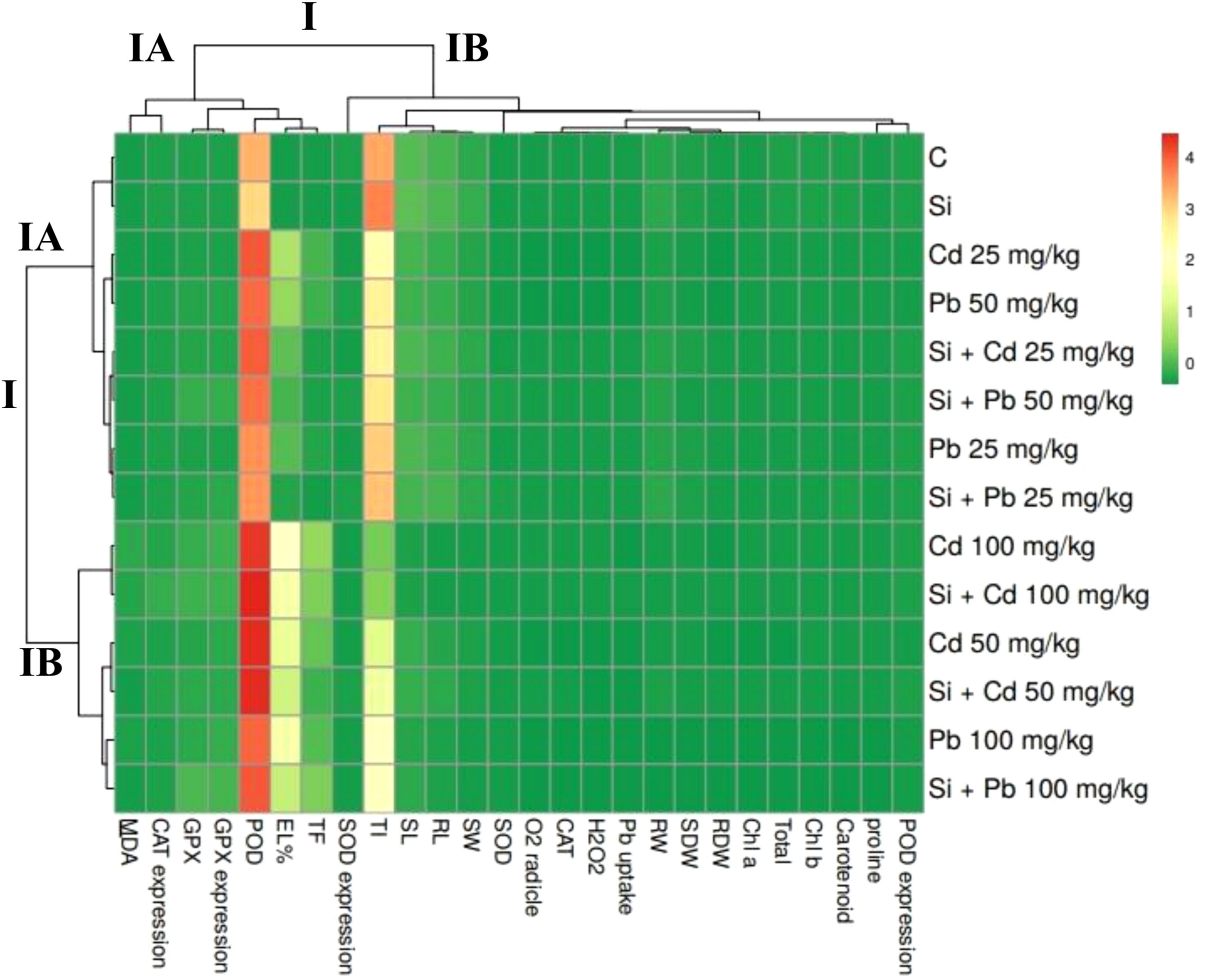
Heat map matrix representing the association between the analysed parameters of chilli plants treated with Cd, Pb and silicon (Si). RL, root length; SL, shoot length; RFW, root fresh weight; SWF, shoot fresh weight; RDW, root dry weight; SDW, shoot dry weight; chl, chlorophyll; EL, electrolyte leakage; H_2_O_2_, hydrogen peroxide; MDA, malondialdehyde; POD, peroxidase; SOD, superoxide dismutase; CAT, catalase; GPX, guaiacol peroxidase; Cd, cadmium; Pb, lead; Si, silicon; TF, translocation factor; TI, tolerance index. Scale bar represents normalized values, uses min-max scaling with negative values indicating lower-than-average and positive values showing higher-than-average. I, represents the main cluster grouping, IA and IB, represents a sub-cluster within cluster I.

**Figure 8:**
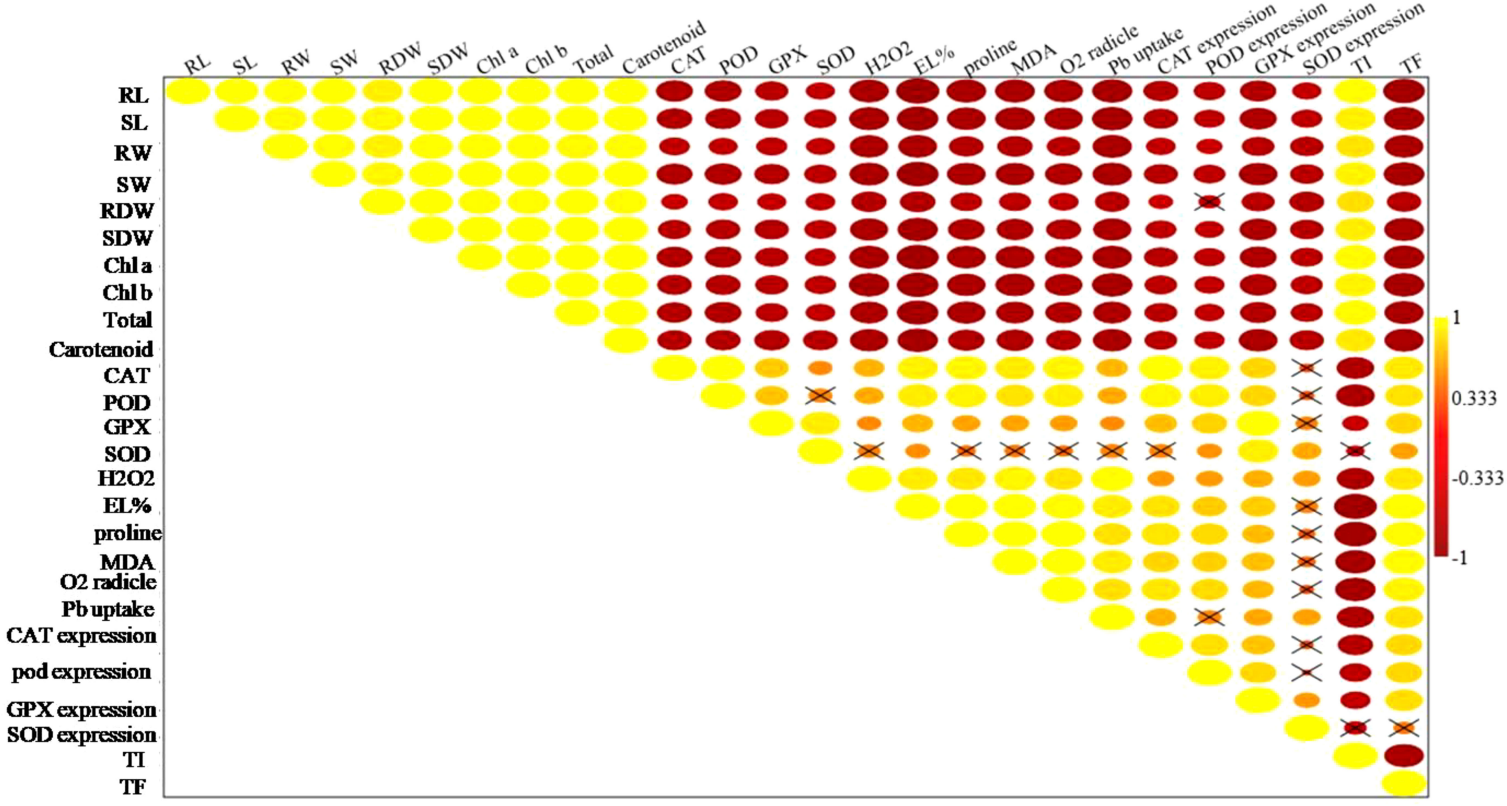
Pearson correlation matrixfor the analysed parameters of chilli plants after Cd and Pb stress and Si treatment. RL, root length; SL, shoot length; RFW, root fresh weight; SWF, shoot fresh weight; RDW, root dry weight; SDW, shoot dry weight; chl, chlorophyll; EL, electrolyte leakage; H_2_O_2_, hydrogen peroxide; MDA, malondialdehyde; POD, peroxidase; SOD, superoxide dismutase; CAT, catalase; GPX, guaiacol peroxidase; Cd, cadmium; Pb, lead; Si, silicon; TF, translocation factor; TI, tolerance index.

**Figure 9:**
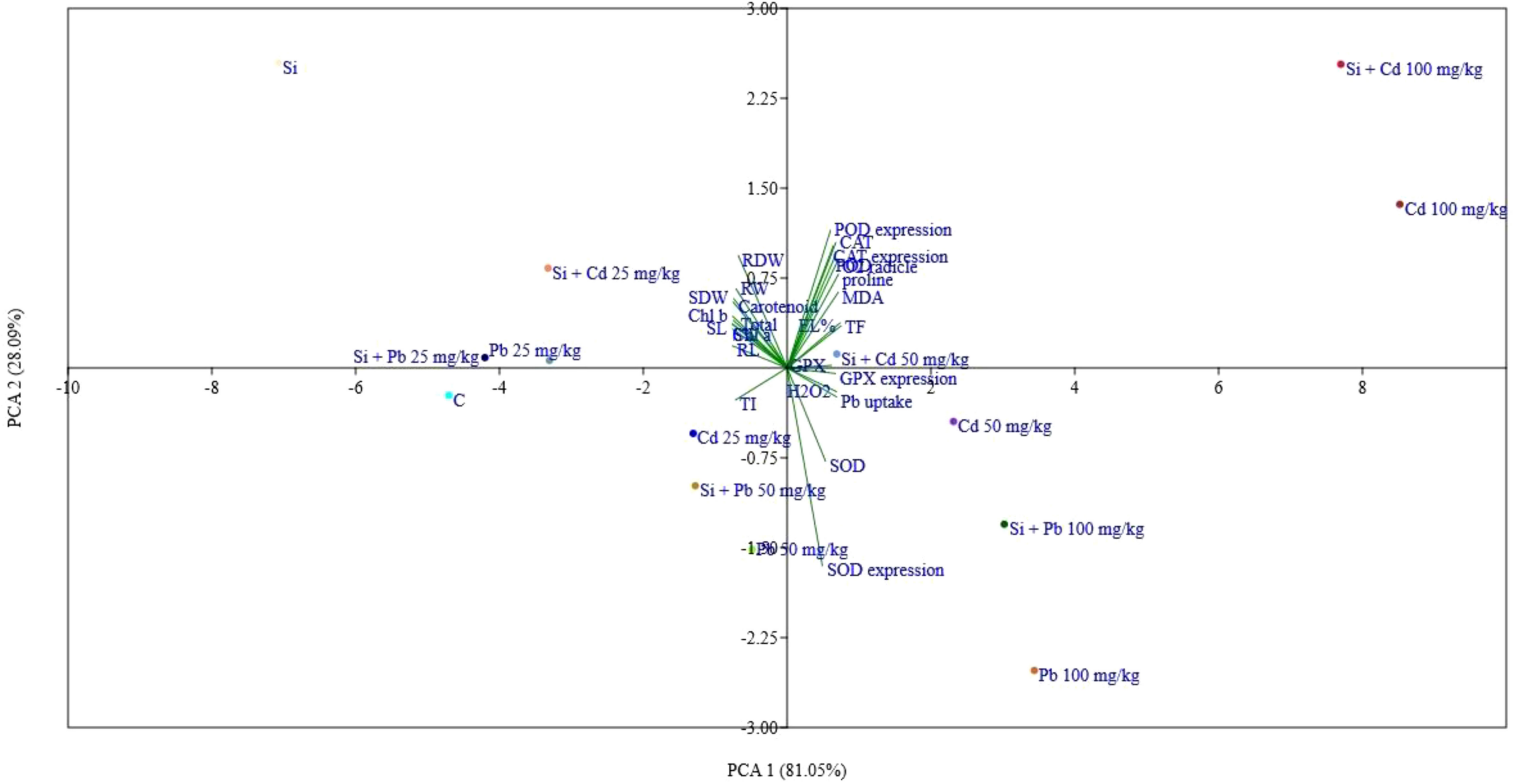
Principal component analysis (PCA) for the analysed parameters of chilli plants treated with Cd and Pb and exogenously applied Si. RL, root length; SL, shoot length; RFW, root fresh weight; SWF, shoot fresh weight; RDW, root dry weight; SDW, shoot dry weight; chl, chlorophyll; EL, electrolyte leakage; H_2_O_2_, hydrogen peroxide; MDA, malondialdehyde; POD, peroxidase; SOD, superoxide dismutase; CAT, catalase; GPX, guaiacol peroxidase; Cd, cadmium; Pb, lead; Si, silicon; TF, translocation factor; TI, tolerance index.

The heatmap illustrates the expression levels of various biochemical parameters in response to different treatments, including control (C), Si, Cd at concentrations of 25, 50, and 100 mg kg^−1^, Pb at the same concentrations, and their combinations with Si (**Figure 7**). Overall, the heatmap reveals the intricate interactions and differential responses of different parameters under various Cd, Pb, and Si treatments (**Figure 8**). The correlation matrix revealed significant positive or negative correlations among all parameters, except for *SOD* enzyme activity and SOD gene expression. In addition to this RDW were non significantly negatively correlated with *POD* gene expression. The PCA biplot reveals the distribution and impact of different treatments on various parameters. PCA 1 explains 81.09% of the variance, while PCA 2 accounts for 8.05% (**Figure 9**). The treatments cluster distinctively, with higher concentrations of Cd and Pb showing significant interactions, indicating increased oxidative stress and changes in antioxidant enzyme activities. Silicon treatments, particularly in combination with Cd, exhibit a mitigating effect, clustering closer to the control and reducing stress parameters. Parameters like CAT, SOD, and GPX expressions, along with MDA levels, show strong associations with the treatments, highlighting the oxidative stress response and protective role of Si. This analysis underscores the effectiveness of Si in alleviating HM-induced stress, ensuring better plant health and physiological stability.

## 4 Discussion

Soil pollution with Cd and Pb is a serious problem as it effects crop productivity and poses a threat to human health. In the past, many studies have shown that Si supplementation improves crop growth and stress resilience during heavy metal stress (Saleem et al., 2022; Puppe et al., 2023). Similarly, in the present study, exogenous application of Si significantly mitigates the adverse effects of Cd and Pb on chilli plants thereby enhancing their vegetative growth and biomass respectively. Si treatment alleviates the toxicity of Cd and Pb-treated chilli plants by improving root and shoot lengths as well as increasing their fresh and dry weights. Cd and Pb at 100 mg kg^−1^ soil significantly reduced root length of chilli plants as compared to control plants which, however, increased/improved when Si was foliarly applied to Cd and Pb-treated plants. The results are consistent with previous studies indicating that Si reduces HM uptake and enhances antioxidant defense mechanisms, thus promoting plant growth under stress conditions (Ahmad et al., 2021; Khan et al., 2021).

The use of Si considerably reduces the negative impacts of Cd and lead Pb on leaf pigments in chili plants, leading to an increase in chlorophyll and carotenoid concentrations. Findings align with previous studies demonstrating Si’s role in enhancing chlorophyll content and photosynthetic efficiency under HM stress. For instance, when applied to arsenic (As)-stressed spinach, Si reduced the metal toxicity and improve the chlorophyll pigments in plants under HM-stressed conditions (Saleem et al., 2023). Similarly, Si increased the Cd tolerance mechanism in cotton plants by improving the photosynthetic machinery and antioxidant enzymes (Farooq et al., 2013; Altaf et al., 2024b).

The HMs triggered a concentration-dependent increase in the activities of CAT. At higher metal dosages (100 mg kg^−1^ soil), the activities of CAT, GPX, POD, and SOD were noticeably elevated. Si application further enhanced enzyme activities under metal stress, compared to their respective controls treated with the same metal concentrations. This enhancement suggests that Si helps bolster the antioxidant defense system, reducing oxidative damage and improving plant resilience under HM stress (Rachappanavar et al., 2024). These findings are similar to previous research indicating that Si enhances antioxidant enzyme activity, thereby mitigating oxidative stress and promoting plant health under adverse conditions (Mostofa et al., 2021).

Under HM stress, Cd and Pb at 100 mg kg^−1^ soil significantly (*p* ≤ 0.05) increased the proline and MDA levels compared to untreated controls. Si application notably decreased these levels, enhancing the plants’ resilience to metal stress. Similarly, Si lowered the MDA levels in metal-stressed plants. These findings align with previous studies, which demonstrate Si’s role in enhancing antioxidant defense, reducing oxidative stress, and improving overall plant health under HM exposure (El-Beltagi et al., 2020; Fatemi et al., 2021). In the current findings the application of Si significantly mitigates oxidative stress in Cd and Pb-stressed chilli plants by reducing electrolyte leakage (EL), hydrogen peroxide (H_2_O_2_), and superoxide radical levels. Nevertheless, the application of Si at lower doses of Cd and Pb significantly decreased these stress markers. This study demonstrated that Si application in chilli plants significantly influenced the uptake of Cd and Pb from contaminated soils. Both Cd and Pb uptake increased with higher metal concentrations, with root tissues accumulating the maximum amounts of metals compared to shoots, consistent with findings from previous studies (Javed et al., 2020). Also, the uptake of Cd, Pb, and Si involves specific transporters and pathways. For instance, Cd is primarily absorbed through the roots via metal transporters such as NRAMPs (Natural resistance-associated macrophage proteins) and ZIPs (Zinc-regulated transporters). Once inside the plant, it is translocated via xylem vessels. Cd uptake can be enhanced by low soil pH and high organic matter (Zhang et al., 2024). Additionally, Pb is absorbed through the root system, primarily through NRTs (Nitrate transporters) and PBS1 (Phosphate-binding site 1). Although Pb mostly builds up in the roots, there is some transfer to the aerial portions. Si is absorbed through the roots via Lsi1 and Lsi2 (Silicon transporters), which are responsible for active uptake and translocation through xylem vessels. Silicon helps mitigate the toxic effects of metals like Cd and Pb. The presence of Si can affect the absorption of Cd and Pb. By generating compounds with Cd and Pb and preventing their transfer to aerial regions, Si can decrease the uptake and toxicity of these metals in chili plants. Additionally, Si strengthens the plant’s defense against oxidative stress brought on by metal toxicity. In plants grown in soils with lower metal concentrations, the uptake of Cd and Pb was reduced, as observed in root tissues containing fewer µg Cd g^−1^ and shoots with lower µg Pb g^−1^ (Zhang et al., 2019). The results suggest that root tissues are more efficient at accumulating metals, which may be a protective response to minimize the translocation to above-ground parts (Podar and Maathuis, 2022). Si application, however, notably reduced metal accumulation in both root and shoot tissues, supporting previous research indicating that Si acts as a mitigator of metal toxicity in plants (Zhou et al., 2021). Specifically, Si reduced Cd uptake in roots by significant percentages (e.g., 25%, 50%, and 100 mg Cd kg^−1^) and in shoots by similar proportions, highlighting its role in enhancing plant resistance to metal stress. This reduction in metal uptake aligns with studies that suggest Si may alter metal transport mechanisms, limiting their translocation to edible plant parts (Zhang et al., 2019). The decrease in Pb uptake with Si treatment further confirms that Si can influence the physiological processes involved in HM absorption and transport (Liu et al., 2015; Ghouri et al., 2024). The observed decrease in metal concentrations in Si-treated plants suggests that Si can be used as a sustainable agricultural practice to reduce the risk of HM accumulation in food crops. Overall, the findings contribute to the growing body of evidence on the beneficial effects of Si in mitigating HM toxicity in plants, with implications for improving food safety in contaminated environments (Yadav et al., 2023).

Increasing concentrations of Cd and Pb in the soil negatively impacted the tolerance index (TI) and translocation factor (TF) of chilli plants. Specifically, Cd at 50 and 100 mg kg^−1^ soil reduced the TI by significant percentages, while Pb at the same concentrations decreased the TI even further, which is consistent with previous findings on the toxic effects of these metals on plant growth and tolerance (Khan et al., 2021). The decrease in TI suggests that high metal concentrations induce stress in the plant, impairing its overall ability to withstand toxic conditions (Bohra et al., 2015). On the other hand, the TF of chilli plants increased with higher Cd and Pb concentrations, indicating greater translocation of metals from the roots to the shoots, a common response in plants exposed to HM stress (Sana et al., 2024). However, the application of Si significantly improved the TI, demonstrating its potential to enhance the metal tolerance of plants under stress (Lai et al., 2023). For example, Si increased the tolerance index in plants exposed to 25 mg Cd kg^−1^ soil by substantial percentages, suggesting that Si plays a role in mitigating the negative effects of metals on plant health. The Si treatment also significantly reduced the TF, particularly for Pb-stressed plants, by decreasing metal translocation from the roots to the shoots (Ngugi et al., 2022). The ability of Si to reduce metal translocation and improve the TI suggests its potential as a natural and sustainable strategy to combat HM contamination in crops (Jan et al., 2024).

Si alone increased *CAT* expression by 1.59-fold, indicating its role in enhancing the plant’s ability to mitigate oxidative stress (Zhou et al., 2021). This finding aligns with previous research suggesting that Si plays a crucial role in reducing the impact of metal toxicity by boosting antioxidant defense systems (Riaz et al., 2023; Song et al., 2009). Cd treatments resulted in a progressive increase in *CAT* expression, with the highest value observed at 100 mgkg^−1^ (3.79-fold), consistent with other studies showing that Cd exposure induces oxidative stress, triggering increased antioxidant activity (Lin et al., 2007). Similarly, Pb exposure also elevated CAT expression, with the highest observed at 100 mgkg^−1^ (2.65-fold), confirming the metal-induced activation of antioxidant responses (Sytar et al., 2013). When Si was combined with Cd, the *CAT* expression increased significantly, especially at 100 mg kg^−1^ of Cd, where the fold increase was 6.78, higher than any other treatment, suggesting a synergistic effect of Si in mitigating the effects of Cd stress (Javed et al., 2020). Si combined with Pb also resulted in increased *CAT* expression, but the effect was less pronounced compared to the Cd treatments, as observed in other studies where Si was found to be more effective against Cd stress than Pb stress (Zhao et al., 2021). The highest *CAT* expression observed in the Si + Cd 100 mg kg^−1^ treatment highlights Si’s potential in alleviating Cd-induced oxidative damage in plants (Ahmed et al., 2023). Regarding the *POD* gene expression, Si treatment alone increased the expression by 1.36-fold compared to the control, supporting its role in improving plant resistance to oxidative stress (Geng et al., 2018). Cd treatments resulted in a progressive increase in *POD* expression, with the highest value (2.45-fold) at 100 mg kg^−1^, which is in line with findings from Pan et al. (2021), who reported that Cd toxicity stimulates the expression of antioxidant enzymes. Pb treatments, however, resulted in lower *POD* gene expression, with the lowest expression observed at 100 mg kg^−1^ (1.21-fold), suggesting that Pb might be less effective in triggering *POD* gene expression compared to Cd (Kohli et al., 2018). Si combined with Cd led to a substantial increase in *POD* gene expression, especially at 100 mg kg^−1^ of Cd, where the expression was 2.59-fold higher than the control, confirming the protective role of Si in enhancing the antioxidant defense system under metal stress (Abd-Allah et al., 2019). Si application also influenced *SOD* gene expression, with a 1.02-fold increase compared to the control. Cd treatments caused a gradual increase in *POD* expression, with the highest expression (2.45-fold) at 100 mg kg^−1^ and *SOD* expression peaked at 1.46-fold at 50 mg kg^−1^ Cd, further emphasizing the role of Si in modulating antioxidant enzyme activities (Abdelaal et al., 2020). Pb treatments resulted in lower *POD* expression (1.21-fold at 100 mgkg^−1^), but the *SOD* expression showed considerable variation, with the highest value (1.98-fold) at 100 mg kg^−1^ Pb, indicating a complex relationship between Pb exposure and the induction of antioxidant enzymes (Limón-Pacheco and Gonsebatt, 2009). Si combined with Pb also increased *POD* gene expression, with a 1.95-fold increase at 100 mg kg^−1^ Pb, while SOD expression ranged from 0.88-fold at 25 mgkg^−1^ Pb to 1.73-fold at 50 mgkg^−1^Pb. This suggests that Si’s effect on *SOD* expression is more variable with Pb compared to Cd, possibly due to the differing mechanisms of metal-induced oxidative stress (Zhou et al., 2021). Overall, these findings confirm that Si plays a critical role in enhancing the expression of antioxidant enzymes, particularly CAT and POD, in metal-stressed plants, providing further evidence of its potential as a sustainable strategy to improve plant tolerance to environmental stressors like HMs (Souri et al., 2021; Eltahawy et al., 2022).

The heatmap in this study illustrates the complex interactions between various biochemical parameters in response to Si, Cd, Pb, and their combinations, revealing significant differences in the plant’s oxidative stress response. The correlation matrix showed that most parameters, except for SOD enzyme activity and gene expression, were significantly correlated, indicating that these parameters are closely linked to the plant’s response to HM stress (Khan et al., 2021). However, the non-significant negative correlation between root dry weight (RDW) and *POD* gene expression suggests that metal stress might affect root growth differently than the antioxidant responses (Labudda et al., 2022). The principal component analysis (PCA) further revealed that Cd and Pb treatments, especially at higher concentrations, led to significant changes in oxidative stress markers, confirming that these metals disrupt plant physiological stability (Zhou et al., 2021). These findings are consistent with previous research demonstrating that Si can reduce oxidative damage in plants exposed to metal toxicity by enhancing antioxidant enzyme activity (Bharwana et al., 2013). The strong association of parameters like CAT, SOD, and GPX with treatments indicates their critical role in managing oxidative stress, with Si playing a key role in boosting these defenses (Kumar et al., 2023).

## 5 Conclusion

The current study demonstrated that exogenous application Si effectively mitigated the phytotoxicity induced by cadmium (Cd) and lead (Pb) in chilli plants, by modulating physiological and biochemical traits. The Si application reduced the uptake of heavy metals (HMs) into plant tissues and enhanced the antioxidant enzymatic activities in chili plants exposed to Cd and Pb, thereby alleviating oxidative stress and toxicity. Furthermore, Si application protected the chillis through mechanisms such as compartmentalization, co-precipitation, and metal chelation within plant organs. This resulted in a reduction in the concentration of free metal ions and a subsequent improvement in plant growth and biomass. Our findings suggest that Si could be an effective strategy for mitigating phytotoxicity in metal-contaminated environments and reducing the accumulation of HMs in chilli seedlings. Further, to fully evaluate the potential of Si in remediating metal-contaminated soils, field validation data are required before recommending their widespread use in field applications. Additionally, the economic feasibility of using different Si sources should be thoroughly assessed to determine the most cost-effective solution for large-scale applications.

## Data Availability

The raw data supporting the conclusions of this article will be made available by the authors, without undue reservation.
