# Supplementary material for: Silicon (Si) foliar treatment modulates Capsicum annuum L. (green chilli) growth and stress responses under cadmium and lead stress
Source: Front Plant Sci. 2025 Jun 13;16:1590148. doi: 10.3389/fpls.2025.1590148 (PMC12202647; doi:10.3389/fpls.2025.1590148)
Supplement: Supplementary file 1 [file DataSheet1.docx]

**Table S1:** The nucleotide sequences of the primers used for qRT-PCR analysis

| Gene name | Gene Bank accession no | Forward primer (5′-3′) | Reverse primer (5′-3′) |
| --- | --- | --- | --- |
| *POD* | XM_016710088 | TTCGACAAGCTGTGCAAACG | CCAGCTAACGCAACAGCATC |
| *SOD* | XM_016720173.2 | ATGAAGCCAAACGGAGGAGG | ACTGCTTCCCATGACACGAG |
| ***CAT*** | NM_001324674.1 | GATTTCTTCTCTTTCCTCC | CGATGTTCCTATTCAATACC |
| ***GPX*** | XM_016717947.1 | GCTAGGGACTCTGTTGTCATTC | GGAGGAGGAATGCTGCTATTG |


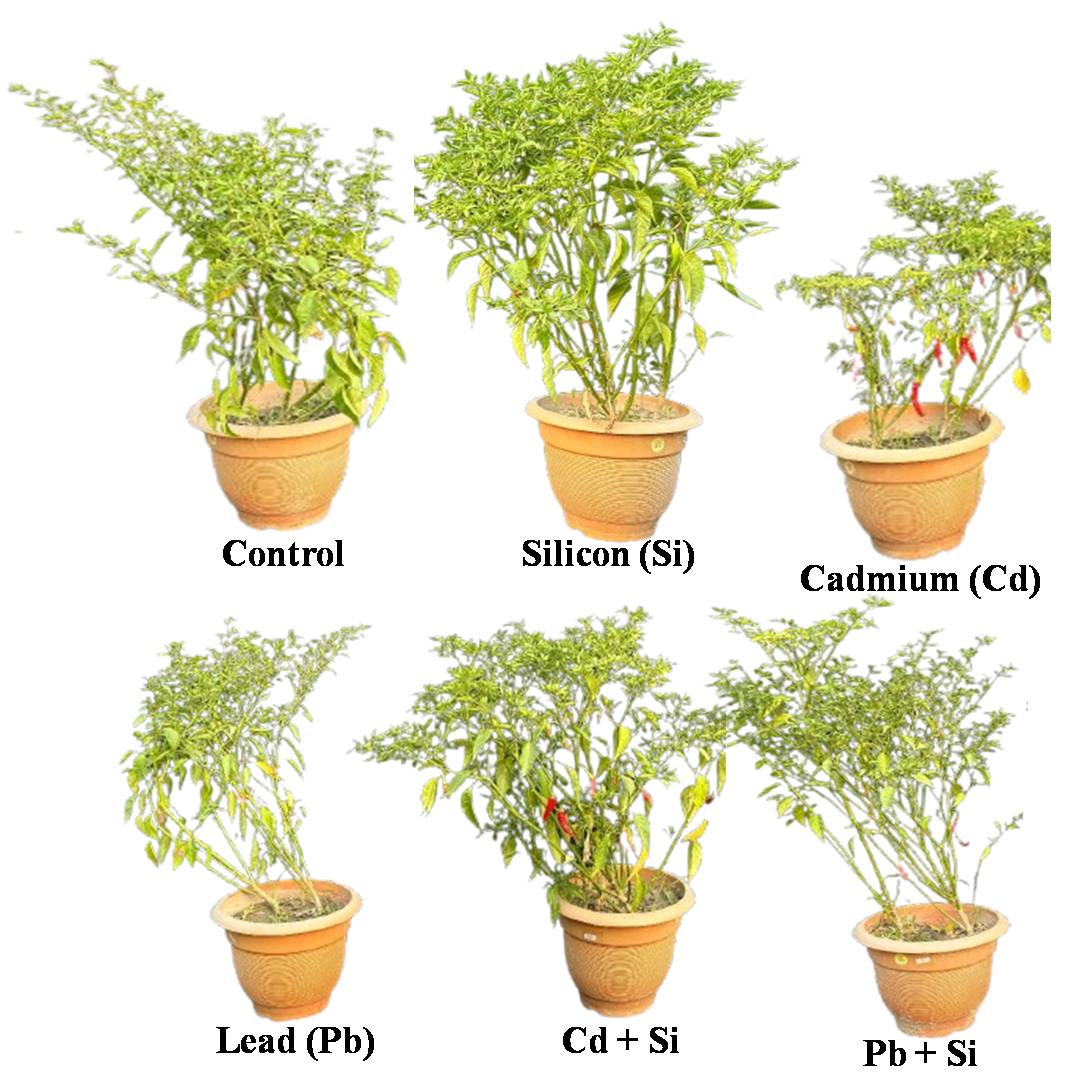


**Figure S1:** Chilli plants grown in soil untreated/treated with Cd, Pb, and applied with Si.
